# Risk of stillbirth in singleton fetuses with advancing gestational age at term: A 10-year experience of late third trimester prenatal screenings of 50,000 deliveries in a referral center in northern Italy

**DOI:** 10.1371/journal.pone.0277262

**Published:** 2023-02-22

**Authors:** Francesco D’Ambrosi, Marta Ruggiero, Nicola Cesano, Matteo Di Maso, Giulia Emily Cetera, Beatrice Tassis, Ilma Floriana Carbone, Enrico Ferrazzi

**Affiliations:** 1 Department of Woman, Child and Neonate, Fondazione IRCCS Ca’ Granda Ospedale Maggiore Policlinico, Milan, Italy; 2 Department of Clinical Sciences and Community Health, Branch of Medical Statistics, Biometry and Epidemiology “G.A. Maccacaro”, University of Milan, Milan, Italy; 3 Department of Clinical Sciences and Community Health, University of Milan, Milan, Italy; University of Palermo: Universita degli Studi di Palermo, ITALY

## Abstract

**Background:**

The risk of intrauterine death (IUD) at term varies from less than one to up to three cases per 1,000 ongoing pregnancies. The cause of death is often largely undefined. Protocols and criteria to prevent and define the rates and causes of stillbirth are the subjects of important scientific and clinical debates. We examined the gestational age and rate of stillbirth at term in a 10-year period at our maternity hub to evaluate the possible favorable impact of a surveillance protocol on maternal and fetal well-being and growth.

**Methods and findings:**

Our cohort included all women with singleton pregnancies resulting in early term to late term birth at our maternity hub between 2010 and 2020, with the exclusion of fetal anomalies. As per our protocol for monitoring term pregnancies, all women underwent near term to early term maternal and fetal well-being and growth surveillance. If risk factors were identified, outpatient monitoring was initiated and early- or full-term induction was indicated. Labor was induced at late term (41+0–41+4 weeks of gestation), if it did not occur spontaneously. We retrospectively collected, verified, and analyzed all cases of stillbirth at term. The incidence of stillbirth at each week of gestation, was calculated by dividing the number of stillbirths observed that week by the number of women with ongoing pregnancies in that same week. The overall rate of stillbirth per 1000 was also calculated for the entire cohort. Fetal and maternal variables were analyzed to assess the possible causes of death.

**Results:**

A total of 57,561 women were included in our study, of which 28 cases of stillbirth (overall rate, 0.48 per 1000 ongoing pregnancies; 95% CI: 0.30–0.70) were identified. The incidence of stillbirth in the ongoing pregnancies measured at 37, 38, 39, 40, and 41 weeks of gestation was 0.16, 0.30, 0.11, 0.29, and 0.0 per 1000, respectively. Only three cases occurred after 40^+0^ weeks of gestation. Six patients had an undetected small for gestational age fetus. The identified causes included placental conditions (n = 8), umbilical cord conditions (n = 7), and chorioamnionitis (n = 4). Furthermore, the cases of stillbirth included one undetected fetal abnormality (n = 1). The cause of fetal death remained unknown in eight cases.

**Conclusions:**

In a referral center with an active universal screening protocol for maternal and fetal prenatal surveillance at near and early term, the rate of stillbirth was 0.48 per 1000 in singleton pregnancies at term in a large, unselected population. The highest incidence of stillbirth was observed at 38 weeks of gestation. The vast majority of stillbirth cases occurred before 39 weeks of gestation and 6 of 28 cases were SGA, and the median percentile of the remaining case was the 35^th^.

## Introduction

Intrauterine death (IUD) at term is a rare but devastating obstetric outcome with a risk that varies from 1.1 to 3.2 cases for every 1,000 pregnancies [1]. The most common causes for IUD include abruption, infection, congenital anomalies, and placental lesions. However, the exact cause of death remains unknown in almost 50% of cases [2, 3]. Interestingly, the respective rate of the various known causes varies according to the median income of the country in which the IUDs have been reported [3].

Several attempts have been made to classify the causes of IUD in the last 50 years. These include the Aberdeen classification, based on clinical history and obstetric findings [4]; the Wigglesworth pathophysiological classification [5]; the relevant condition at death (ReCoDe); a hierarchical classification of death-related conditions [6]; and the Causes of Death and Associated Conditions (CODAC) classification [7]. The classification of causes and risk factors is expected to promote the targeted prevention of IUD. Moreover, the identification of risk factors is crucial for preventing IUDs. If we restrict the gestational age of intrauterine fetal death to term gestation, post-term gestational age per se becomes a recognized risk factor [8, 9]. Accordingly, obstetric guidelines recommend the induction of labor at late term, no later than 41^+6^ weeks [10–12]. Late fetal growth restriction is a reported cause within an array of known and unknown causes. Another relevant risk factor for IUD is impaired fetal growth. In particular, birth weights below the 5th centile and above the 95th centile are both at a higher risk of fetal demise [13]. This condition is typically the result of poor placental efficiency at and near term. However, as per epidemiological surveys it is not usually included among the surveillance protocols that are followed for births at term.

The aim of the present study was to assess the rate of IUD at term (after 37^+0^ weeks of gestation) in a referral maternity center during the last decade, where a screening protocol for fetal growth assessment was systematically adopted during the late third trimester.

## Materials and methods

In this retrospective study, we consecutively included all women with singleton pregnancies that gave birth at our obstetric unit at the Fondazione IRCCS Ca’ Granda Ospedale Maggiore Policlinico, Mangiagalli Maternity Centre, from January 1, 2010 to December 31, 2020. Data on IUDs were collected from the Birth Certificate (CEDAP) database, an official digital repository that reports all relevant clinical data for each birth in Italy. These dates were cross-examined with the original clinical record at admission and then with registry of the mortuary of our institute, where each IUD from 24 weeks of gestation is mandatorily recorded and classified; a lack of recording can lead to a criminal charge to the responsible officials. As expected, the three sources of data proved coherent recordings of these severe events. The gestational age at admission of patients with IUD was carefully assessed, with a preference for the first-trimester ultrasound when available, as described later in this section.

From the whole ten years cohort, we excluded patients who delivered preterm (before 37^+0^ weeks), those who had multiple pregnancies, and those who had pregnancies with fetuses with known chromosomal or structural abnormalities as recorded on the dedicated digital repository (Astraia Software, version 2.3.1, GMBH, Munich, Germany) adopted for antepartum surveillance of maternal and fetal data since 2010.

Since 2010, our referral maternity center has established a screening protocol for antepartum surveillance during the late third trimester for women who were planning to give birth at our institution. Gestational age was determined by a first-trimester ultrasound examination [14], as it is routinely performed as part of standard care according to the Italian National Health Service (NHS) guidelines [15], or very rarely by means of a second trimester ultrasound fetal biometry if the previous criteria were not satisfied [16, 17].

Between January 2010 and December 2016, the screening protocol was started at 37^+0^–38^+0^ weeks of gestation. During the examination, we recorded the maternal and obstetric history, body mass index, brachial blood pressure and the presence of lower extremity edema. When one of these indices was abnormal, a urine test strip was additionally performed.

An obstetric sonologist performed a transabdominal ultrasonographic scan (Voluson, Voluson E8 Expert, GE Medical Systems, Milwaukee, WI, USA, Samsung HERA W9) to evaluate the fetal presentation, site of placental insertion, fetal heart rate and movements, the umbilical pulsatility index, and the amniotic fluid index. Fetal growth was not routinely assessed at the time of the first evaluation; however, we recorded the last fetal biometry performed during the third trimester according to the Italian NHS guidelines [15]. Fetal biometry was assessed in cases of gestational diabetes, hypertensive disorders, and a previous poor obstetric history. Moreover, fetal growth was assessed using ultrasound in overweight and obese women, who presented with a fundal height that was difficult to estimate.

Since January 2017, our protocol has been modified as follows: the first screening evaluation was anticipated at 35^+0^–37^+0^ weeks of gestation. Fetal growth and weight estimation [18] were systematically added to the sonographic examination. The middle cerebral artery pulsatility index and the cerebroplacental ratio were added in 2019 and the uterine artery pulsatility index was added in 2020.

After the first assessment, subsequent examinations were scheduled at 39^+0^ and 40^+3^ weeks in patients who planned to deliver vaginally at our maternity center.

Between 2010 and 2016, patients were admitted for labor induction at 41^+0-+1^ weeks of gestation if they had not yet delivered. From 2017 onwards, women younger than 40 y received further evaluation at 41^+0^ weeks and eventually underwent late term induction of labor at 41^+2+4^ weeks. Pregnant women older than 40 y were offered induction of labor at 40^+2-+3^ weeks of gestation.

Patients planning to deliver by cesarean section for obstetrical, medical, or psychological indications received the first evaluation alone and were subsequently scheduled for abdominal surgical delivery at 39^+0+3^ weeks of gestation.

Pregnant patients that screened positive for obstetrical complications such as hypertensive disorders, gestational diabetes treated with insulin therapy, severe cholestasis, anhydramnios, late small for gestational age (SGA) (abdominal circumference or fetal weight below the 10^th^ centile), or large for gestational age (LGA) (abdominal circumference or fetal weight above the 90^th^ centile) were scheduled for a dedicated protocol of surveillance and early- or full-term induction of labor.

Cases involving premature rupture of membranes (PROM) were treated according to the local protocol: prompt induction for cases with a positive group B streptococcus (GBS) vagino-rectal swab or laboratory signs of inflammation white blood cell count ≥ 15×100/μL, C-reactive protein ≥ 1.5 mg/dL or clinical signs of chorioamnionitis [19], and expectant management with induction after 24 h in all other cases.

For each week of gestation, we computed the number of spontaneous labors, induced labors, and antepartum elective cesarean sections.

We then identified all cases of stillbirth during this ten-year period. A double-check of the CEDAP certificate was performed on the registry of the mortuary of our institute, where each IUD from 24 weeks of gestation is mandatorily recorded and classified; a lack of recording can lead to a criminal charge to the responsible officials. The gestational age at admission of patients with IUD was carefully assessed as previously described, with a preference for the first-trimester ultrasound when available.

For each IUD, we collected data from routine diagnostic investigations to assess the cause of death, including fetal autopsy, histological examination of the placenta, fetal and placental microbiological swabs, maternal screening for thrombophilia, and autoimmune diseases. To classify the causes of death, we used the following criteria for the CODAC classification [7]: 0: infectious deaths caused by infections affecting the mother or intrauterine structures; 3: congenital anomalies—chromosomal anomalies and structural malformations; 4: fetal conditions, diseases, and events; 5: cord conditions, diseases, and events; 6: placenta—conditions, diseases, and events of the placenta and membranes; 7: maternal conditions, diseases, and events; 8: unknown, unexplained, and unclassifiable causes of death.

Data were analyzed using the statistical package IBM SPSS (version 22.0; IBM, New York, NY, USA). Categorical variables are expressed as absolute frequencies (number of women per group) and percentages, whereas continuous variables are expressed as means and standard deviations (SD). The intrauterine fetal mortality rate was calculated by dividing the number of IUDs by the total number of women considered in the study. We then estimated the risk of stillbirth for each week of gestation (incidence per week) by dividing the number of stillbirths observed that week by the number of women at risk in the same week. For a given gestational age, we defined women at risk of stillbirth as those who were still pregnant at the beginning of the week minus half the number of women who delivered that week [20].

A distribution model was used to calculate the 95% confidence interval (95% CI) of the overall rate of IUD and the relative risk per week of gestation. The fetal weight percentile of IUD cases was calculated according to the Italian national newborn weight percentile table for sex, parity, and week of gestation [21].

This study was approved by the Institutional Review Board of Fondazione IRCCS Ca’ Granda, Ospedale Maggiore Policlinico, Milan, Italy (reference n. 0052088 27 december 2021). This is a retrospective study conducted on medical records. Patients provided written informed consent to use the data.

## Results

**Fig 1:**
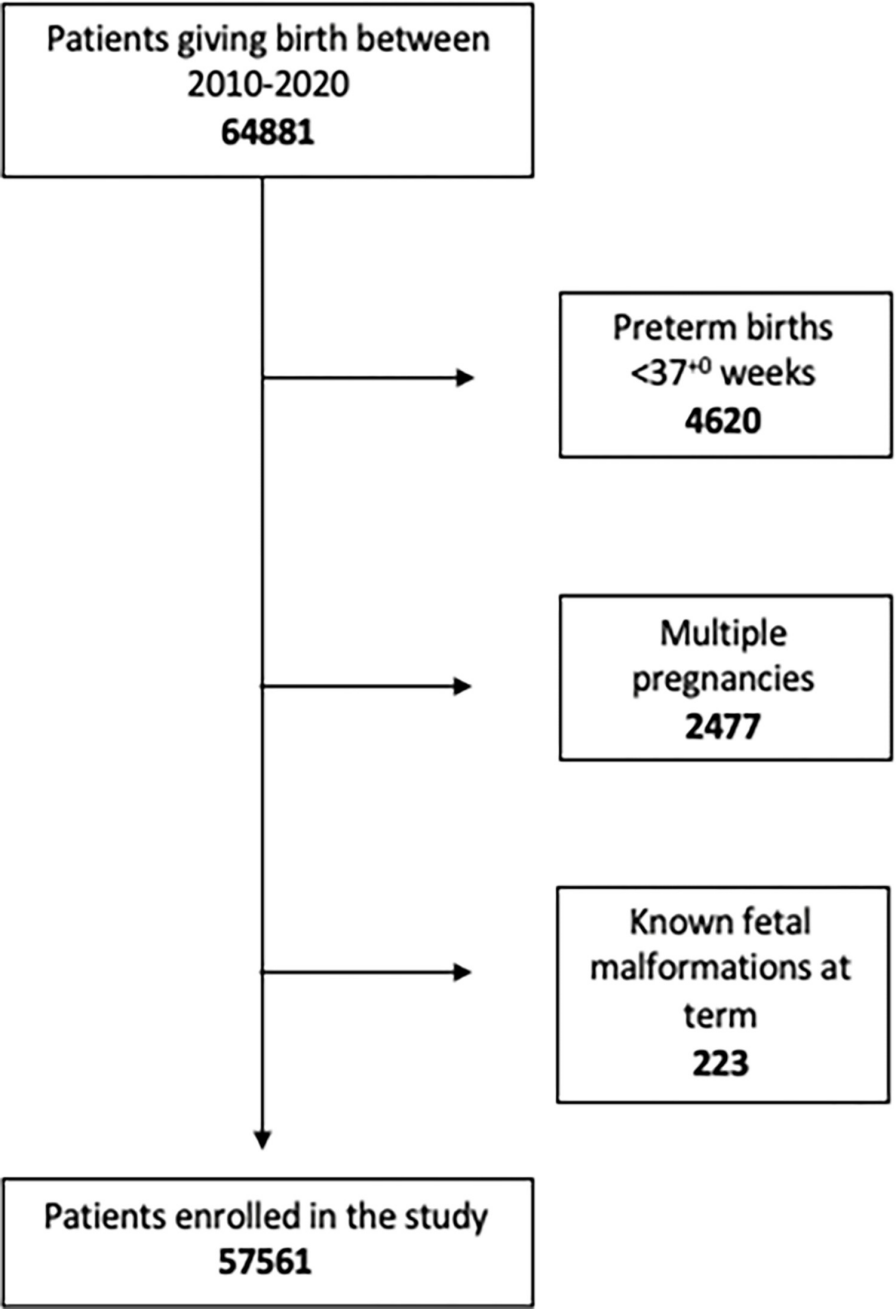
Clinical study design.

During the study period, a total of 64,881 women gave birth at our referral maternity hospital. We excluded 4,620 (7.1%) women who gave birth before 37+0 weeks of gestation, 2,477 (3.8%) women with multiple pregnancies, and 223 pregnant women that presented with fetal abnormalities and delivered at term (0.3%) (Fig 1).

Finally, a total of 57,561 (88.7%) pregnancies were analyzed in the present study. Among these, 305 (0.5%) women did not undergo any obstetric evaluation or ultrasonographic scan during pregnancy; however, in this subgroup, coincidentally, no cases of IUDs had occurred.

**Fig 2:**
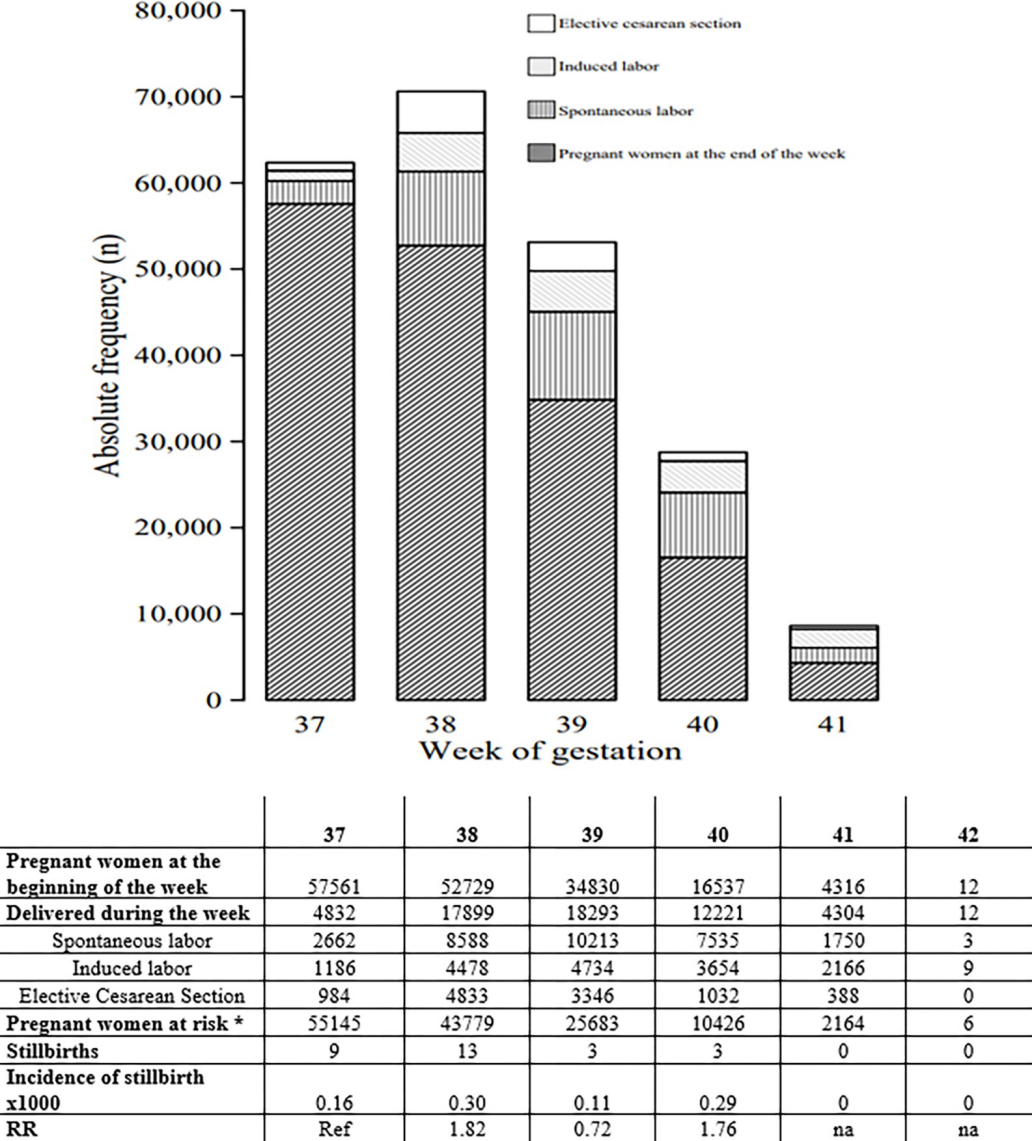
Number of cases per week of gestation according to clinical management: Spontaneous labor, cesarean section prior to labor, induced labor, expectant management.

Of the 57,561 women at term, 10,583 (18.4%) underwent antepartum cesarean section, 30,751 (53.4%) were admitted for spontaneous labor, and 16,227 (28.2%) were induced according to the local protocol at 41^+0+3^ weeks, except for nine cases of late referral at 42 weeks that were induced at admission (Fig 2).

In this large single-center consecutive cohort, we observed 28 cases of IUD at term, resulting in an overall term mortality rate of 0.48 per 1000 pregnant women (95% CI: 0.3–0.7). The same mortality rate was observed from January 1, 2010 to December 31, 2017 (17 IUDs occurred in 34,683 pregnancies) and from January 1, 2017 to December 31, 2020 (11 IUDs in 22,573 pregnancies), when the new protocol for maternal and fetal surveillance was introduced.

The incidence of IUD per week of gestation is presented in Fig 2. The highest incidence occurred at 38 weeks of gestation (0.30 per 1000 pregnant women, RR 1.82). Interestingly, only three out of 28 fetuses died in utero after 39^+3^ weeks of gestation.

The maternal and fetal characteristics of IUD cases are summarized in Table 1.

**Table 1:** Maternal and fetal characteristics of intrauterine death (number of cases, mean and standard deviation or median and interquartile range, where appropriate).

Characteristics	Stillbirth (28)
**Maternal**	
Age (year)	34.4 (5.2)
<20 years	0
>40 years	4
BMI (Kg/m^2^)	27.8 (3.9)
Multiparous	9
In vitro fertilization	2
Symptoms at admission	
Asymptomatic	18
Reduced fetal movements	8
PROM	1
Symptomatic uterine contractions	1
**Fetal**	
Gestational age at time of diagnosis	38^+1^ (37^+5^–38^+5^)
Weight at birth (gr)	3037 (2702–3221)
Weight at birth (percentile)	35 (17,5–57)
Small for gestational age	6
Intrauterine growth restriction	3
Large for gestational age	2
Placental weight (g)	480 (420–510)

The causes of IUD according to the CODAC classifications [7] for the whole cohort and more specifically for the six cases of SGA (<10th centile,) [21] are presented in Table 2. The cause of fetal death in eight cases remains unknown.

**Table 2:** Causes of intrauterine death in the whole cohort and in small for gestational age fetuses.

Causes of death	All cases (n)	SGA (n)
*Unknown*	8	0
*Placental conditions*	8	1
*Cord conditions* *	7	2
*Infection*	4	3
*Congenital anomalies*	1	0

(*) three cases with more than 3 neck loops, two cases with 2 tight cord neck loops 1 true cord knots, one marginal cord insertion

The following maternal complications and risk factors were discovered among women with intrauterine fetal death: the presence of heterozygous factor V Leiden mutation in three women (histological examination of these placentas revealed the presence of extensive intervillous thrombi in two cases and cord thrombosis associated with four cord loops around the neck in one case), hypertensive disorders of pregnancy well controlled by therapy in two women (one case of abruption), maternal cocaine abuse in one woman, and placental abruption without hypertension in one woman. One out of three patients with the V Leiden mutation refused labor induction at 39 weeks of gestation and was admitted seven days later with fetal IUD.

Out of the total 28 women with IUD, six did not participate in the late third-trimester surveillance protocol. Among them, two had an undiagnosed SGA fetus, one had an umbilical cord knot, and the other three did not have any known risk factor for IUD.

**Fig 3:**
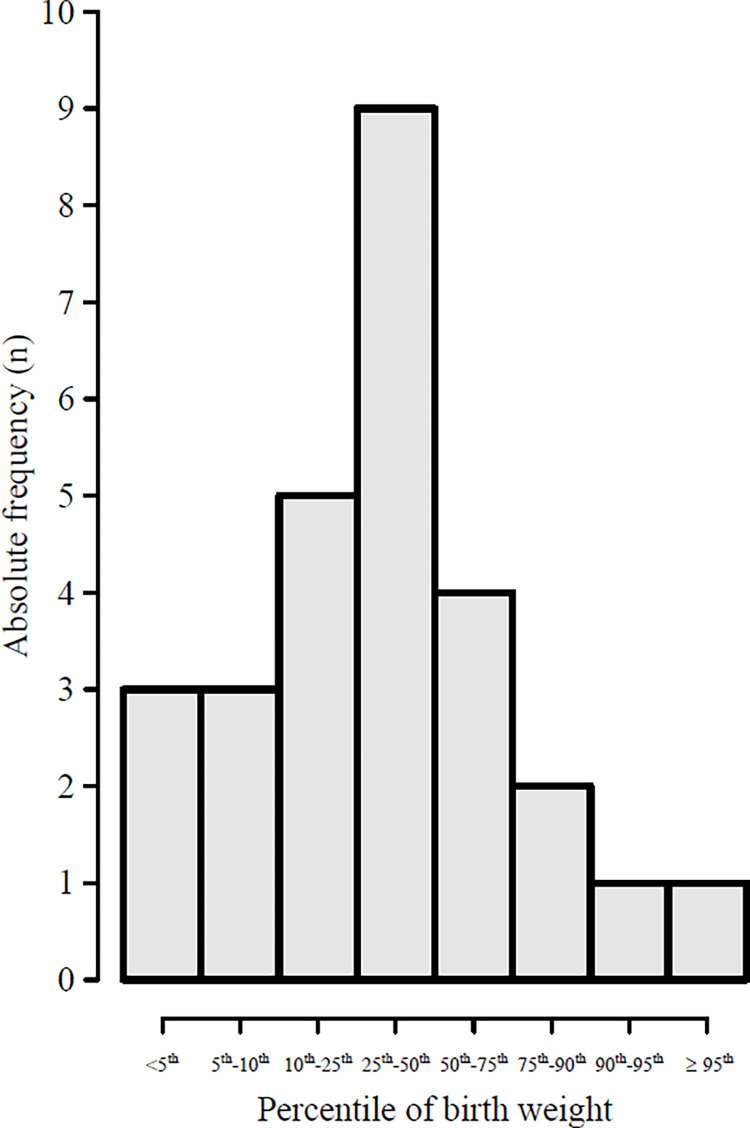
Birth weight percentile of cases of intrauterine death.

Fig 3 shows the percentile distribution of the 28 cases of IUD. Of the 28 cases, 20 newborns presented with a weight that was below the 50th percentile.

## Discussion

In the present large single-center retrospective study, we found an overall rate of IUD at term for singleton pregnancies that did not belong to the low-risk criteria, to be as low as 0.48 per 1000 pregnant women. In this large cohort, 22 of the 28 IUD cases occurred before 39+0 weeks of gestation, and the highest weekly incidence of IUD was observed at 38 weeks of gestation.

Six cases of undiagnosed SGA were found among the stillbirths, while the other appropriate for gestational age (AGA) cases were mostly due to unpredictable and unpreventable causes, such as cord knots.

The rate of IUD in our cohort is significantly lower than that reported in previous literature, which ranges from 1 per 1000 to 3 per 1000 pregnant women [1, 8, 10, 22–24], although it is difficult to compare our data with that of other studies due to the differences in study populations, socioeconomic background, prevalence of minority ethnic groups in the referral area, and local clinical criteria adopted for maternal fetal monitoring at term and for induction.

In a population-based survey conducted by Dongarwar et al. [23] on 117 million non-selected term pregnancies from 1982 to 2017 in the United States, the IUD rate was 2 per 1000 pregnancies. In the study, the authors concluded that extensive research would help identify women at risk during early pregnancy, and that robust risk stratification strategies are required to optimize the monitoring and intervention for decreasing the IUD rates in the United States.

In a similar recent population-based study conducted in the Netherlands, Kortekaas et al. [24] analyzed the data from the Perinatal Registry of Netherlands, including a cohort of 479,097 singleton pregnancies at term between 2010 and 2012. The overall rate of stillbirths was 0.92 per 1000 pregnancies (440/479,097), of which 85% occurred at 37^+0^ to 40^+6^ weeks.

Previous studies have compared the outcomes of cases induced at term versus expectant management by means of large retrospective cohorts [25], systematic reviews and meta-analysis of retrospective cohorts [1], or a review of prospective trials comparing expectant management versus induction of labor [9]. In a large retrospective study, Knight et al. [25] used the English Hospital Episode Statistics to analyze 77,327 nulliparous pregnant women over 35 y of age at full term (beyond 39^+0^ weeks of gestation). Induction of labor at 40 weeks of gestation was observed in 33.1% of cases and was marginally higher than the 28.2% rate observed in our study; notably, the cohort considered only women aged more than 35 y. In the expectant management group (51,744), stillbirths occurred in 175 cases (3.38 per 1000), whereas in the induction group (25,583) has been reported 8 cases of stillbirths and the rate was 0.31 per 1000. However, the study did not include stillbirths occurring at 37–38 weeks of gestation, since the aim of the study was to prove that “routine induction of labor at or after 39 weeks of gestation reduces the risk of perinatal mortality in first-time mothers aged 35 y or older compared with expectant of spontaneous labor. Consequently, the possible burden of IUD occurring at early term, which is a time window when a significant number of IUDs might occur, may have been missed.

In contrast, while almost half of our study population was younger than 35 y, we included women delivering at early term, a gestational age at which the highest number of stillbirths occurred. If we were to exclude early term stillbirths, the overall mortality rate in our study would have been 0.17 per 1000 pregnant women.

To date, the largest reported study on this topic is the systematic review and meta-analysis by Muglu et al. [1] which included 15 million pregnancies from 13 studies on low- and mixed-risk pregnancies. The stillbirth incidence per week, calculated using the same formula that we adopted, increased from 0.39 per 1000 pregnancies at 37 weeks to 0.47 at 38 weeks, 0.63 at 39 weeks, 0.99 at 40 weeks, and 1.62 at 41 weeks. The overall mortality rate was 1.4 per 1000 pregnancies, which is higher than that observed in the present study and can be attributed to the pooled week-specific risks determined using a multilevel (studies and women), mixed-effects logistic regression model without covariates and with random intercepts. Muglu et al. [1] concluded that compared to 40 weeks, when pregnancies continue up to 41 weeks, there is a 64% increased risk of IUD, with one additional IUD occurring for every 1,449 pregnancies. The discussion of this large cohort pointe out that “Some of these apparently ‘low risk’ pregnancies may also have had undetected fetal growth restriction. But continuation of such pregnancies to term is in line with current practice, where there is no routine ultrasound monitoring of fetal growth”.

In our study, we observed only three cases of fetal demise that occurred beyond 40 weeks of gestation, with one additional IUD occurring every 4,903 pregnancies that continued beyond 40^+0^ weeks of gestation.

The Cochrane review by Middleton et al. [26] included 34 randomized controlled trials (RCTs), involving 21,500 women with mostly low-risk pregnancies. The trials compared a policy of inducing labor at 41 completed weeks of gestation (> 287 days) with a policy of expectant management. The risk of IUD at term was reported to be 2 per 1000 in ongoing pregnancies in the expectant management arm and 1 per 1000 pregnancies in the induction arm (RR 0.30, 95% CI 0.12–0.75), with a significantly better Apgar score in the induction arms. However, of the three largest RCTs by Hannah et al. [27], Wennerholm et al. [8], and Grobman et al. [28], only the ARRIVE trial by Grobman et al. [28], supported the hypothesis of universal induction at 39^+3^, whereas the other two only provided evidence of a reduction in the number of stillbirths by induction at late term. The external validity of the ARRIVE trial has been debated due to the very young age, prevalence of obesity beyond 50%, and low socioeconomic status of the recruited population [29]. However, no significant differences in perinatal death (0.1‰) were observed between the two arms of the study.

Different populations and their relative neonatal anthropometric charts must be taken into consideration to elucidate the association between fetal estimated weight and IUD. It is now universally accepted that SGA fetuses have an increased risk of perinatal death [13, 29–32]. Poon et al. [30] considered a population of 113,019 live births and 437 stillbirths (4‰) after 24 weeks of gestation in single phenotypically normal fetuses. The SGA rate among stillbirths was 23.2%. Early identification of SGA fetuses is of major importance in minimizing IUD rates at term. Notably, despite our efforts, few cases could not be detected by our surveillance protocol, and six fetuses (21%) among the stillbirths were SGA. Two out of the six did not undergo our screening protocol, while the other four missed detection on ultrasound screening. These cases occurred before 2019, when our screening protocol did not include the evaluation of the middle cerebral artery pulsatility index, the cerebroplacental ratio and the uterine artery pulsatility index. These interrogations might be of help in sorting out otherwise”normal” fetuses to be monitored and possibly induced at early term or term, but not simply managed expectantly. From 2019, we did not observe undetected SGA cases among IUDs. In addition, we should consider that recent diagnostic criteria for late intrauterine growth restriction (IUGR) also include a fall in growth of more than 50 percentiles, even within the normal reference values [31, 32]. Among the 28 IUDs, the mean weight was at the 35^th^ percentile; therefore, we might have underestimated late fetal growth problems.

Hypertensive disorders of pregnancy are also known risk factors for stillbirth [33, 34]. Despite our protocol of early induction in these cases, we observed two cases of IUDs in patients with hypertension apparently well controlled by therapy, occurring respectively at 37^+3^ weeks and 38^+2^ weeks of gestation.

In a relevant proportion of cases (29%), the cause of IUD remained undefined; this rate is consistent with that reported in the literature, which varies from 30% to 60% [2, 3, 35, 36]. When a plausible cause of death was identified in our series, it was mostly unpredictable, such as umbilical knots or loops, placental thrombosis, chorioamnionitis, or placental abruption. These causes are particularly difficult to detect during routine ultrasonographic or clinical examinations and are not strictly related to gestational age.

A potential limitation of our study is its single-center design, which could cause bias owing to local attitudes and skills. The strength of our study lies in the large sample size and study design aimed at capturing real-life data on the universal adoption of a standardized screening protocol for prenatal surveillance. In this regard, the retrospective design should be viewed as an advantage because the data were not biased by any prospective trial-positive effects [37].

Our findings, although not achieved by means of a randomized trial, support the efficacy of strict surveillance of maternal and fetal well-being and growth at term in identifying fetuses who would benefit from an induction of labor due to maternal-fetal complications and in selecting women with an uncomplicated pregnancy who may be induced at late term without an additional risk of IUD.

In conclusion, a screening protocol for prenatal surveillance in the late third trimester seems to effectively reduce the incidence of stillbirth by applying labor induction to selected cases at risk of fetal demise or obstetrical complications. Twenty-two of the 28 cases of IUD occurred in the early term, with the highest weekly incidence of IUD at 38 weeks of gestation. This near-term maternal and fetal clinical and sonographic screening might represent a safe alternative to universal induction at full-term, while this policy will require almost 50,000 inductions to prevent six of the 28 cases observed. The cost efficacy of a screening protocol with multiple outpatient clinic examinations, versus the longer hospital stay of induced pregnancies and the possible higher rate of cesarean sections in induced labors [25], should be assessed.
